# Neurodevelopmental disorders—high-resolution rethinking of disease modeling

**DOI:** 10.1038/s41380-022-01876-1

**Published:** 2022-11-25

**Authors:** Konstantin Khodosevich, Carl M. Sellgren

**Affiliations:** 1 Biotech Research and Innovation Centre (BRIC), Faculty of Health and Medical Sciences, University of Copenhagen, 2200 Copenhagen, Denmark; 2 Department of Clinical Neuroscience, Centre for Psychiatry Research, Stockholm Health Care Services, Stockholm County Council, Karolinska Institutet, Stockholm, Sweden; 3 Department of Physiology and Pharmacology, Karolinska Institutet, Stockholm, Sweden

**Keywords:** Psychiatric disorders, Neuroscience, Biological techniques

## Abstract

Neurodevelopmental disorders arise due to various risk factors that can perturb different stages of brain development, and a combinatorial impact of these risk factors programs the phenotype in adulthood. While modeling the complete phenotype of a neurodevelopmental disorder is challenging, individual developmental perturbations can be successfully modeled in vivo in animals and in vitro in human cellular models. Nevertheless, our limited knowledge of human brain development restricts modeling strategies and has raised questions of how well a model corresponds to human in vivo brain development. Recent progress in high-resolution analysis of human tissue with single-cell and spatial omics techniques has enhanced our understanding of the complex events that govern the development of the human brain in health and disease. This new knowledge can be utilized to improve modeling of neurodevelopmental disorders and pave the way to more accurately portraying the relevant developmental perturbations in disease models.

## Introduction

**Fig. 1:**
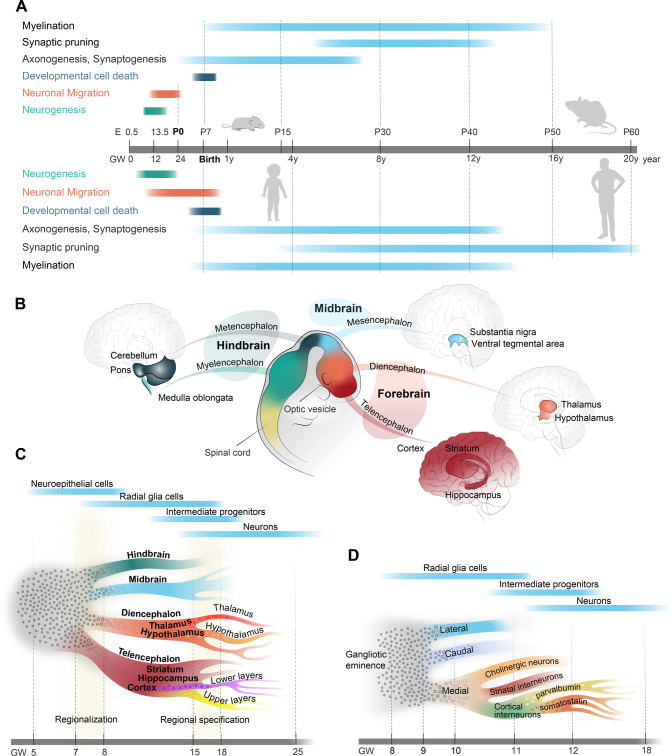
Overview of brain development. **A** The schedule of major developmental processes during brain development in mice (upper part) and humans (lower part). **B** Anatomical correspondence between developing brain regions in the early embryonic brain and the postnatal brain. **C** Schematic representation of differentiation strategy during brain development, starting from undifferentiated neuroepithelial cells (gray) to more differentiated radial glia cells, intermediate progenitors, and neurons. The timeline in gestational weeks (GW) is not-to-scale. Subtype differentiation within brain regions (such as upper/deeper layer principal neurons in the cortex or different neuropeptide-specific neurons in the hypothalamus) is organized rather orthogonally to areal signatures (such as rostro-caudal differentiation) and have strong temporal dependence. For instance, in the cortex, deeper layer principal neurons are produced as early as GW9, whereas switch to upper layer principal neurons happens at GW14-16. Colored cells that appear before the actual branching indicate that the cell fate is primed, mainly by epigenetic mechanisms. **D** Schematic representation of differentiation strategy for the ganglionic eminence that generates telencephalic inhibitory neurons. The medial ganglionic eminence is expanded to show an example for differentiation into classes, families, and subtypes of neurons.

Brain development is a tremendously complex process in which a myriad of neuronal and non-neuronal cell types is generated and assembled into functional circuits in a highly organized manner. Neurodevelopmental disorders (NDDs) are common brain disorders, which usually have a major impact on the affected patients and their families and arise when developmental processes are perturbed by various genetic and environmental factors [1–7]. Given the complexity of human brain development and the variety of possible perturbations, the clinical presentations and suspected disease etiologies are very heterogenous within the NDD group. Historically, the NDD concept has been restricted to disorders in which perturbations are thought to occur during embryonic brain development, e.g., as for autism spectrum disorder, attention deficit hyperactivity disorder, and intellectual disability. However, with a better understanding of the maturational processes continuing up to early adulthood (Fig. 1A), disorders with a later age of onset, e.g., schizophrenia, are now also referred to as NDDs.

In the last decade, our understanding of the brain development at the cellular level has been deepened by technological breakthroughs in single-cell and spatial omics. Combined with more precise methods for genetic engineering, improved disease models, and the growing number of genetic variants found to be associated with disease, we are now much better positioned to identify biological mechanisms causing NDDs. Nonetheless, great experimental and conceptual challenges lie ahead and will require careful considerations. In the current update, we summarize emerging new concepts in our understanding of the developing human brain and discuss how these advances could be implemented in NDD models.

## The developing human brain in high resolution

Adult brain regions develop from embryonic brain vesicles, which are organized along an anterior-posterior axis of the neural tube (Fig. 1B). The telencephalon and diencephalon (forebrain regions) give rise to the cortex, striatum, and hippocampus, and the thalamus and hypothalamus, respectively. The mesencephalon (midbrain region) instead gives rise to the superior part of the brainstem, including structures such as the substantia nigra and ventral tegmental area; while the metencephalon and myelencephalon (hindbrain regions) give rise to the cerebellum and pons, and the medulla oblongata, respectively.

Recent developments in single-cell and spatial omics have revolutionized our understanding of the cellular diversity across regions and time periods in the developing human brain (Fig. 1C, D). Generally, early neural stem cells in embryonic brain, neuroepithelial (NE) cells, transform into more differentiated radial glia (RG) cells, which in turn differentiate into intermediate progenitor cells and neurons (Fig. 1C). Single-cell analysis of first-trimester human brain tissue have revealed that region-specific transcriptomic signatures first appear in NE and RG cells at gestational week (GW) 7–8, whereas prior progenitors are highly similar across developing brain regions [8] (Fig. 1C). Regional transcriptomic signatures expand over late first and second trimester, peaking into regional specification at GW15–18 [9, 10], when regions are being further divided into specific areas (Fig. 1C).

Cortex area-specific signatures can be detected at ~GW17–18 [9–11] (Fig. 1C), as a rostro-caudal gradient without clear borders [9, 10]. These areal transcriptomic signatures can already be identified in progenitors [9–11], which are likely primed by a few transcription factors and preassembled epigenetic mechanisms. Such priming in progenitors triggers large transcriptomic specification programs in early postmitotic neurons, with further differentiation taking place during migration and the early stages of neuronal maturation [12–14]. Importantly, specification of laminar position of cortical neurons (i.e., upper to lower layers) is organized orthogonally to areal signatures (i.e., anterior to posterior). Lamination specification also has a strong temporal dependence, where newborn, lower-layer neurons can be identified as early as GW9, switching to mainly upper layers between GW14 and 16 [12].

Cell-type specifications have also been studied in other brain regions, such as the hippocampus and hypothalamus (Fig. 1C). In the hippocampus, common progenitors of excitatory neurons are specified into dentate gyrus and nondentate gyrus neurons at GW16–18, followed by further specification associated with morphogenesis (GW20–22) and functional maturation (GW25–27) [15]. In the hypothalamus, specific transcriptomic signatures for hypothalamic nuclei can be distinguished at GW7–8, while nucleus diversification and onset of specific neuropeptide expression occurs at GW18–25 [16].

A number of studies have implicated inhibitory neurons in NDDs [2, 3, 17, 18]. Telencephalic inhibitory neurons are born in the transitory ganglionic eminences (GEs), which are subdivided into three regions—the medial, caudal, and lateral eminences (MGE, CGE, and LGE, respectively) (Fig. 1D). The MGE and CGE produce mainly cortical, striatal, and hippocampal interneurons, and LGE produces mainly striatal projection neurons and olfactory bulb interneurons. Similarly to principal neurons, telencephalic inhibitory neuron progenitors are noncommitted and primed to be specified during transition from precursors to neurons [19, 20]. However, it seems that stratification of neuronal cell fate is expedited for inhibitory neurons and individual subtypes can be distinguished in each GE already at GW11–18 [15, 19–21], for example, up to 11 subtypes of somatostatin neurons in MGE [20].

Developmental trajectories for non-neuronal cells have been also studied at high resolution. Oligodendrocyte precursor cells (OPCs) are generated via Pre-OPC states from common neural progenitors [22, 23] and the first wave of OPC generation is observed already at GW8–10 [23]. Microglia instead arises from erythromyeloid progenitor cells in the yolk sac and can be detected in the developing brain from at least GW9 [24]. Microglia are a highly heterogenous cell population during the fetal period and display temporal gene expression changes [24].

To date, the third trimester and the long postnatal period of the developing human brain have only been sparsely studied by single-cell analyses, mostly due to limitations in the availability of clinical samples. This means that while we have good resolution atlases for neurogenic, cell migration, early differentiation, and morphogenesis stages, data are sparse for the long and important maturational period of human brain development.

## From developmental trajectories to modeling neurodevelopmental perturbations

When a risk factor for a NDD is first identified in epidemiologic or genetic association studies, the question arises as to where in the brain and during which developmental period(s) the relevant consequences for disease take place. Simply put, when, where, and how does risk transform into perturbation? Ideally, the initial modeling approach, before any certain mechanism is prioritized, should be broad with complex readouts for all brain regions, developmental stages, and cell types. For practical reasons, studies are, however, usually focused on a specific brain region, cell type, or developmental stage. Even if such decisions are based on prior knowledge, bias can easily be “inherited” as previous studies could have selected neuronal subtypes or developmental periods, for instance, according to available samples and tools. A good example is parvalbumin (PV) interneurons in the cortex: almost every study of a NDD evaluates an impact on PV interneurons, which might give the impression that PV interneurons are the major contributors to all NDDs. However, recent studies show that other types of interneurons, various types of excitatory neurons, and glia might be equally affected, and the unbiased view by single-cell analyses of postmortem human tissue reveals complex transcriptomic changes affecting multiple neuronal subtypes and glia [25, 26].

**Fig. 2:**
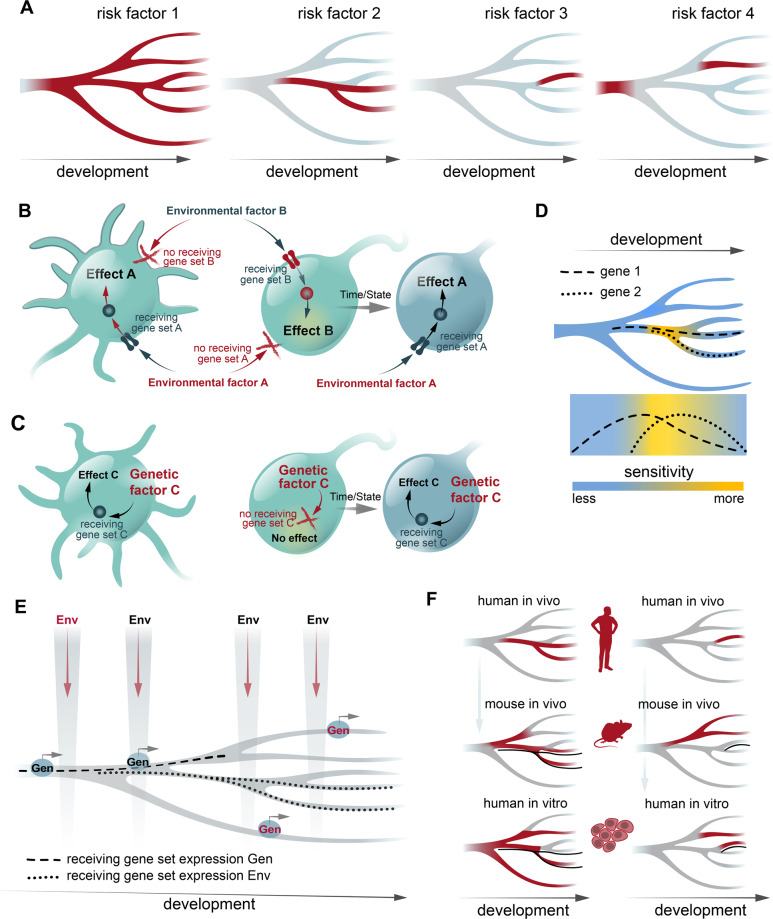
Modeling neurodevelopmental perturbations. **A** Examples of risk factor gene expression overlaid on developmental trajectories during cell differentiation and maturation, from neural stem cells on the left differentiating towards committed cell types on the right. Red denotes gene expression. **B**, **C** The concept of selective vulnerability to environmental and genetic risk factors, based on receiving gene sets, i.e., gene sets that define cellular components (as signaling network) that are necessary to respond to a particular risk factor, as first described in [6]. The response to genetic risk factors originates from within the cell, whereas for environmental risk factors the initial response is transmembrane. The susceptibility to a risk factor can change over the course of development due to changes in expression of receiving gene sets, illustrated by the cells on the right. **D** Sensitivity to risk factors over development is based on ability to respond to the risk factors. A combinations of two genes that constitute a receiving gene set is shown (dotted lines—gene expression). **E** Modeling strategies on what to model, and on what not to model. If a genetic (Gen) risk factor/receiving gene set is expressed only during certain period/cell lineage (dashed line), it makes sense to model within this period (black but not red Gen label). Similarly, for an environmental risk factor, only period when receiving gene set is present (dotted lines) is relevant to model (black but not red Env label). **F** Assessment of conservation of risk factor/receiving gene set expression across models. Left and right columns show examples when mouse in vivo or human in vitro models, respectively, are better in mimicking human in vivo.

Fortunately, recent advances in our understanding of brain development by single-cell omics have improved our precision and could guide modeling strategies. By utilizing developmental trajectories, we can identify at single-cell level, when and where a gene is expressed (Fig. 2A). Hence, using reconstructed developmental trajectories, it can be determined for each gene in which brain regions, developmental periods, cell types, and differentiation stages this particular gene is expressed. For a given neurodevelopmental perturbation, we can then select a gene or a combination of genes that code for factors that are required to respond to a given perturbation, i.e., receiving gene sets [6] (Fig. 2B, C). For a genetic risk factor, a receiving gene set is then centralized around the primarily affected gene. For an environmental risk factor, complexity increases as an environmental factor can reach the cell via multiple routes. For instance, in case of maternal inflammation during pregnancy, the overall impact on a given cell in the embryonic brain is a combination of individual impacts of each pro-inflammatory molecule that reaches this brain cell [6]. The complexity is further augmented by secondary receiving gene sets, when microglia is directly activated by maternal inflammation and then in turn impacts surrounding neuronal cells. Using receiving gene sets, we can predict cells that should respond to the perturbation. As cell-state transcriptional signatures are dynamic and form a gradient throughout development, especially in the case of uncommitted progenitors [8–10], this suggests that there is a gradient sensitivity to risk factor exposure during development (Fig. 2D). Since gradient sharpness increases over the course of development from first commitment to regionalization and subtype differentiation, early developmental periods might have a greater regional sensitivity, which then changes during later developmental periods to areal- and cell type-specific sensitivity. Nonetheless, even in the adult stage, gradient gene expression between differentiated cell types and transitory cell states remains [27]. From the perspective of receiving gene sets with multiple regional and temporal gene functions, the plethora of clinical presentations for polygenic NDDs is then hardly surprising. This often requires additional phenotypic specification, preferably using quantitative traits, although the approach is still compatible to cross-disorder designs.

Implementing developmental trajectories to assess the impact of developmental perturbations has an immense impact on a modeling strategy, i.e., what to model and what not to model (Fig. 2E). Thus, when a certain genetic factor has been proposed to be involved in a NDD and the gene is not expressed in principal neurons, but rather is expressed in GABAergic ones and maybe even in a certain subtype, modeling and analyses should initially be focused on this subtype. Similarly, for the developmental period, if the gene is expressed only in immature neurons and not in progenitor cells, then modeling should be initially focused on immature neurons. For environmental factors, modeling is more complex as there is typically a lack of prior knowledge for how an environmental factor reach the cell and triggers the response. Furthermore, there might be several routes for triggering the response. For instance, several individual soluble molecules that mediate environmental factor response might activate different transmembrane receptors in the cell. A combination of all receptors and their intracellular signaling pathways comprises a receiving gene set for the cell. Whereas it seems difficult to model an overall impact of an environmental factor, individual components can be readily modeled, e.g., such as IL-6 and IL-17 response in maternal inflammation [28, 29].

Such simple considerations could provide tremendous help and promote more efficient use of our resources. As an example, implementation of single-cell data to understand the mechanisms underlying the impact of the 15q13.3 microdeletion, a genetic risk factor for several psychiatric disorders [30], revealed a hidden impact on embryonic development, suggesting that a reduction in *KLF13* expression in progenitors might be the major neurodevelopmental driver of the 15q13.3 microdeletion phenotype [3]. Importantly, although a considerable amount of work is still left to complete reconstruction of developmental trajectories, enough data have been generated to start predicting with good precision the impact of many neurodevelopmental perturbations.

Finally, conservation of developmental trajectories is also important to consider in regard to the model being used, typically in vivo animal models versus in vitro human models (Fig. 2F). For instance, if expression patterns along developmental trajectories for a risk factor are much different between humans and rodents, a mouse model of this risk factor might be a poor choice for reproducing the impact of such a perturbation in humans, and then a human cellular model or primate model should be considered instead. A concrete example is the component 4 (*C4*) genes. Here, detailed genotyping of the most strongly associated GWAS locus in schizophrenia has revealed that copy numbers of the isogene *C4A* explains most of the risk, while *C4B* copy numbers do not increase schizophrenia risk [31]. However, rodents lack specialized *C4* genes, and it was first shown in human in vitro models that the unique link between *C4A*, excessive synaptic pruning, and schizophrenia could be established [4]. Another study showed similar data using a humanized mouse model, confirming in vivo that C4A binds synapses more efficiently than C4B, while overexpression of *C4A* leads to an increased microglial engulfment of synaptic structures [32].

Non-human primates (NHPs) offer an alternative strategy to model human NDD risk factors during brain development. Although there are a number of limitations in implementation of NHP models, such as long breeding periods, difficulties in genetic manipulations, and ethical considerations, advantages over rodent models are greater similarity in gestational time and physiology, brain architecture [33–35], cell type-specific signatures [36] and developmental trajectories [35, 37]. It is also worth noting that NHP, similarly to humans, exhibit a large outer subventricular zone—a region of developing brain that is rudimental in rodents and was proposed to have the major contribution to the large expansion of the cortex in primates [38]. Additionally, while genetic risk modeling is limited in NHPs, modeling environmental risk factors is rather feasible [39, 40] and allows to study developmental processes that are absent in rodents. Thus, NHP models help bridging rodent data with pathological processes in humans and enhance translatability of rodent results.

Overall, recent data from single-cell analyses show both considerable rodent-to-human conservation of cell-type specification as well as human-specific mechanisms, emphasizing that a dual approach using animal models as well as human cellular and NHP systems is required for modeling NDDs. Below, we discuss the latest progress in in vivo animal and in vitro human modeling of NDDs and propose how knowledge of developmental processes can be implemented in these strategies.

## Improved modeling of NDDs: in vivo animal models

In recent years, the translational potential of animal modeling in NDDs, including psychiatric disorders, has been the subject of debate, and challenges as well as opportunities in this regard have been widely discussed elsewhere, see, for example [41]. We anticipate that while it might be difficult to model schizophrenia or autism in animals, it is feasible to model specific perturbations that are risk factors for NDDs, which will help us identify impaired developmental mechanisms that cause changes in neuronal network assembly and behavioral phenotypes relevant for NDDs. Thus, below we focus on how new knowledge of developmental processes can be implemented to design improved in vivo models of neurodevelopmental perturbations in rodents.

**Fig. 3:**
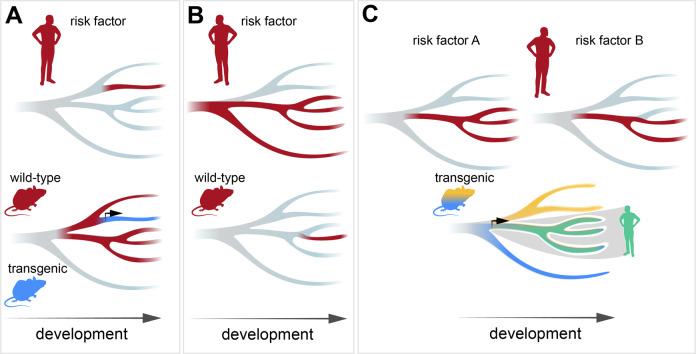
Strategies for modeling neurodevelopmental perturbations in rodents. **A** When a risk factor is present in a specific cell lineage in the human brain (in red, top), but the expression is broader in the rodent brain (in red, bottom), utilization of cell type-specific transgenics can restrict risk factor analysis to the cell lineage relevant for human brain (in blue). **B** When the expression of a risk factor is broader in the human brain (in red, top) than in an animal model of interest (in red, bottom), the model can be utilized to target specific cell lineages or developmental periods. **C** To model an interplay of two risk factors (in red, top), transgenic models with intersectional gene expression can be utilized (bottom, blue and yellow are individual transgenic lines, the intersection is in green).

One of the key benefits of the available high-resolution data for brain development is that it allows for precision in modeling. In general, comparative studies report relatively good similarity in specification patterns during development of human and mouse brains [15, 20, 42]. Thus, the main features of developmental trajectories are conserved, such as prominent brain-region transcriptional signatures [43], largely orthogonal transcriptional signatures for spatial and temporal specification [44], noncommitted progenitors and first-commitment signatures for neuronal subtypes detected postmitotically [45–47], and priming progenitors via epigenetic mechanisms [42, 45, 46]. Nevertheless, notable differences between human and mouse developmental trajectories have been reported, such as human-enriched/-specific populations of progenitors [8] and neurons [48], or the ability of some progenitors to generate both excitatory and inhibitory neurons [49]. Therefore, we propose that when we aim to understand how a developmental perturbation affects “human” brain development, the credibility of a rodent model for this particular perturbation should be assessed first. If a risk factor (or receiving gene sets as responders to a risk factor) shows a very different developmental expression pattern between humans and an animal model of interest, the feasibility of using this type of animal model for modeling “human” developmental perturbation should be scrutinized. Nevertheless, even in those situations when there are significant differences in expression, advantages in recombinant technology in animal models enable us to “focus” an animal model on modeling the human situation. For instance, when a risk factor is present in a specific cell lineage in human brain, but its expression is broader in the animal model, then utilization of cell type-specific transgenics or turn on/off gene expression can restrict risk factor analysis to the cell lineage relevant for human brain (Fig. 3A). In contrast, when the expression of a risk factor is broader in human brain than in an animal model of interest (multiple examples of such cases have been identified [36, 50, 51]), the model can be utilized to target specific lineages or developmental periods (Fig. 3B). Importantly, rich resources in animal transgenics allow for multi-factor modeling by intersectional and/or substructional approaches [52, 53]. For instance, when two risk factors are present in overlapping, but different developmental period(s) or developmental cell lineage(s), the risk factor interplay can be modeled by intersection of specific promoters, including potential temporal dimension by turning on/off promoters (Fig. 3C). Finally, pooled CRISPR screens with single-cell RNA-sequencing readouts, such as Perturb-seq, can be applied to in vivo models [54] and include thousands of genetic perturbations across coding regions [55]. This allows for modeling interactions between a large number of genetic risk factors as well as interactions between genetic risk factors and environmental perturbations. Perturb-seq has already been implemented to study how multiple NDD genetic risk factors located in coding regions influence the developing mouse brain [54]; and with the possibility to target CRISPR screens at non-coding regions [56], Perturb-seq also allows for investigating NDD risk variants located in non-coding regions.

## Improved modeling of neurodevelopmental disorders: human in vitro models

**Fig. 4:**
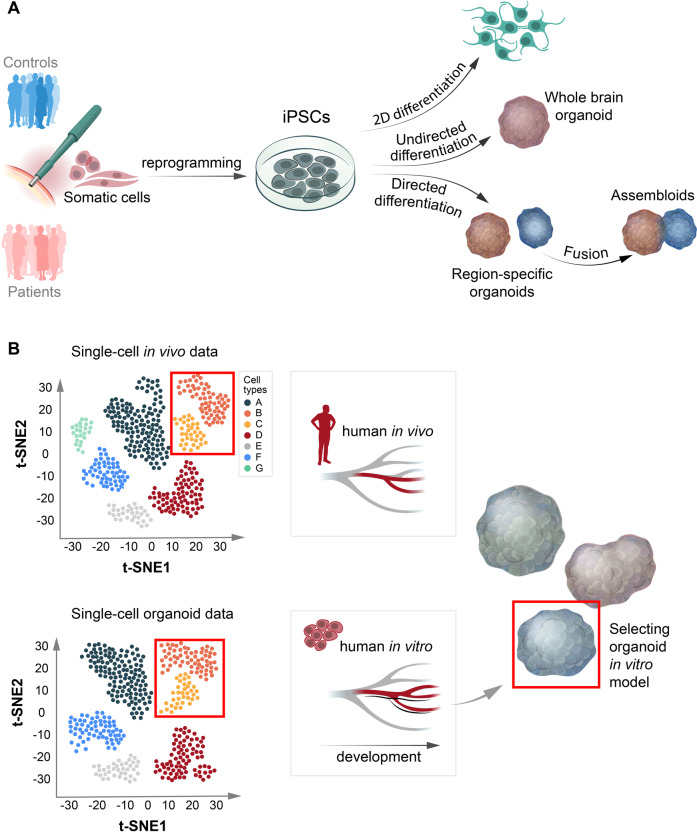
Strategies for modeling neurodevelopmental perturbations in human brain organoids. **A** Overview of different strategies to model neurodevelopmental perturbations in vitro in human cellular systems, including brain organoids. **B** When an organoid model is deemed adequate for modeling a neurodevelopmental perturbation, careful consideration should be made concerning the protocol that is most suitable for the given research question instead of applying a “one size fits all” approach to organoid modeling. Single-cell and spatial omics datasets are then a crucial resource that can help us evaluate the protocol that is most adequate for modeling a given developmental perturbation.

The ability to reprogram human somatic cells to induced pluripotent stem cells (iPSCs) has become a valuable tool in NDD modeling. Early efforts to differentiate patient-derived neural-like cells in monolayer have rapidly been complemented by models that use cellular 3D aggregates to recapitulate the tissue architecture of the developing human brain [57] (Fig. 4A). By supporting more complex cell-cell interactions and cell diversity while reducing artificial interactions with plastic surfaces, these approaches are likely to more adequately mimic human brain development, both in transcriptomic signatures and cell-type composition. Nonetheless, brain organoids are not “mini-brains” and can only approximate the developing brain in vivo. Data for developmental trajectories from single-cell omics can then help to evaluate each in vitro 3D modeling strategy for a given developmental perturbation. As in vitro 3D models display limitations in regard to developmental stages, cell types, and/or brain regions, the expression pattern of a risk factor (or receiving gene sets as responders to a risk factor) across cell types/states, brain regions, and developmental stages should be assessed in vivo to select those that are relevant for modeling (Fig. 4B).

A growing number of protocols currently exist and can broadly be divided into undirected approaches, generating whole-brain organoids (relying more on the self-organizing capacity of pluripotent stem cells in 3D aggregates), and directed approaches to generate brain region-specific organoids (emphasizing more the external patterning factors to support intrinsic cues). Single-cell omics characterizations have been applied to many of the existing protocols and have revealed important information about how a protocol covers cellular diversity and differentiation states during development [58–63]. For most protocols, comparing organoid gene expression with in vivo references suggests that the maturation process typically reaches at least the second trimester. Nonetheless, directed protocols that culture cortical organoids over longer time periods suggest that early postnatal developmental stages can be reached at around 300 days in vitro [59], thus largely mimicking in vivo maturation. In contrast to a previous report [64], upregulation of cellular stress pathways in these organoids also remain relatively flat during differentiation and comparable to datasets generated from the fetal cortex [59]. In a recent systematic comparison of different organoid protocols using available single-cell transcriptomic datasets [58], genes related to endoplasmic reticulum were upregulated in both directed and undirected protocols. This suggests that despite using different technical approaches with orbital shaking or spinning bioreactors in vitro, the exchange of oxygen and nutrients into the core of the organoids is still compromised and needs to be taken into account. In support of this finding, transplanting human organoids into cortices of living mice markedly reduced stress-marker expression [64].

In comparison to primary tissue, organoids also show less defined subtypes in general and some cell types are represented in lower numbers (e.g., oligodendrocytes) or are absent (e.g., microglia) [65]. Especially the lack of microglia has obstructed organoid utility given their important regulatory roles in neurodevelopment [66]. By further reducing neuroectoderm stimulation, mesoderm-derived progenitors have, however, been shown to emerge spontaneously in undirected organoids and display microglia markers at later time points [67]. Single-cell transcriptomic profiling of such microglia-like cells at later stages has also shown a convincing clustering with fetal microglia [68]. Given the general limitations of less guided protocols, such as more pronounced organoid-to-organoid variability and relatively low and variable microglia counts, other approaches have also been proposed, such as adding mesodermal yolk sac progenitors to patterned brain region-specific organoids [69] or adding iPSC-derived microglia to undirected organoids [70]. Furthermore, as an alternative to undirected whole-brain organoids, fusing of brain region-specific organoids to form so-called assembloids has also been performed and applied to NDDs [71].

In summary, we propose that when an organoid model is deemed adequate for modeling a NDD, careful consideration should be made concerning the strategy that is most suitable for the given research question instead of applying a “one size fits all” approach to organoid modeling. The accumulated single-cell omics data will then be a useful resource to select the best strategy according to the most relevant cell types, brain region(s), and developmental stage(s) for a given developmental perturbation. Similarly, the experimental design needs to be carefully selected: is a case-control study more adequate or are isogenic lines preferable? In vivo validation should also be considered, either by using animal models or by observational clinical studies.

## Limitations of the current state of single-cell analysis and future directions

Although the single-cell technology has progressed massively over the last few years and led to immense advance in understanding of human brain development, we are still far from complete multi-modal reconstruction of developmental trajectories. So far, only single-cell RNA-sequencing has reached a very high resolution, up to 10–12,000 genes per cell for technologies like Smart-seq [72], whereas other omics modalities at the moment are at lower resolution. For instance, current state-of-the-art for single-cell proteomics limits detectability to ~1000 of most abundant proteins per cell [73, 74], and there is a lack of methods to measure posttranslational modifications at single-cell level. Resolving these bottlenecks will be of major importance for the field as proteins regulate cellular functions, and mRNA and protein content for a given gene do not always match. Capturing of mRNA or protein context is another general limitation, as even for mRNA most protocols capture only 1–5% of total mRNA content. Additionally, single-cell analysis of brain tissue, whether at single-cell or single-nucleus level, involves rigorous tissue processing, which destroys neuronal/glial processes and the information about axonal and dendritic mRNAs is lost. Spatial transcriptomics has emerged as a method with subcellular resolution, e.g., MERFISH [75, 76], that allows for both detection of mRNA distribution in cellular compartments (including axons and dendrites) and large-scale brain tissue architecture reconstruction. Finally, neuronal connectivity can be also reconstructed at single-cell level by a combination of single-cell omics with cell-cell interaction [77].

In this review, we have updated the perspective of NDD modeling such that greater emphasis should be placed on the available single-cell omics data from primary tissue and disease models in selecting an appropriate modeling strategy. Combined approaches including human cells and in vivo conditions should be prioritized. A number of large-scale atlases are available for studying developmental trajectories at single-cell resolution (e.g., via Human Cell Atlas [78] or BRAIN Initiative—Cell Census Network [79]), and further initiatives have been established to study diseases at such resolution (e.g., LifeTime [80]). Nevertheless, classification of cell types still varies from dataset to dataset (although some initiatives try to deal with this problem [81]), number of individual samples is limited, interindividual variability is largely unstudied, and only some developmental periods are covered. Finally, while many studies have been conducted for embryonic development, postnatal development that include developmental cell death [82, 83] and maturational processes related to functional specification [84, 85] have not been studied as comprehensively in single-cell analyses, due to limitations in availability of material for this developmental period in tissue banks. Paradoxically, this is the key period for (1) understanding how the phenotype in many NDDs, including major psychiatric disorders, is built during maturation and network assembling, and (2) identifying drug targets and a therapeutic window. Studies targeting postnatal maturation would be tremendously important for the field and accelerate our understanding of the mechanisms driving NDDs.
